# Early Visual Perception Potentiated by Object Affordances: Evidence From a Temporal Order Judgment Task

**DOI:** 10.1177/2041669516666550

**Published:** 2016-09-09

**Authors:** Atsunori Ariga, Yuki Yamada, Yusuke Yamani

**Affiliations:** Hiroshima University, Japan; Kyushu University, Japan; Old Dominion University, VA, USA

**Keywords:** affordance, temporal order judgment, perception/action, object recognition

## Abstract

Perceived objects automatically potentiate afforded action. Object affordances also facilitate perception of such objects, and this occurrence is known as the affordance effect. This study examined whether object affordances facilitate the initial visual processing stage, or perceptual entry processes, using the temporal order judgment task. The onset of the graspable (right-handled) coffee cup was perceived earlier than that of the less graspable (left-handled) cup for right-handed participants. The affordance effect was eliminated when the coffee cups were inverted, which presumably conveyed less affordance information. These results suggest that objects preattentively potentiate the perceptual entry processes in response to their affordances.

Daily activities involving manual interaction with objects require accurate perception as well as selection and execution of appropriate actions. According to Gibson (1979), each object affords specific actions, such as holding a right-handled coffee cup (i.e., a cup whose handle is on its right side) with the right hand, and such action in turn facilitates further perception of the object in visual environments. *Object affordances* can thus shape actions for the active perception of the object of interest (Gibson, 1979). Previous research has demonstrated that object affordances facilitate manual responses (Cho & Proctor, 2010; Pappas, 2014; Tucker & Ellis, 1998) as well as identification of objects (Adamo & Ferber, 2009; McNair & Harris, 2014; Riddoch, Humphreys, Edwards, Baker, & Willson, 2003; Roberts & Humphreys, 2011), which is known as the *affordance effect*.

For example, Tucker and Ellis (1998) asked participants to judge whether graspable objects (e.g., a frying pan) were upright or inverted while manipulating the mapping of a response hand to object (upright-inverted and left–right) orientations factorially. First, responses to upright objects were generally faster than responses to inverted objects regardless of their response hand. Second and importantly, although the horizontal left–right orientation of objects was task irrelevant, responses were faster when they were made by the afforded hand than the nonafforded hand. These results suggest that object affordance information automatically triggers relevant motor responses after the registry of the objects in the visual system (e.g., Adamo & Ferber, 2009; Craft & Simon, 1970; Michaels, 1988; Tucker & Ellis, 1998; Wallace, 1971).

However, Anderson, Yamagishi, and Karavia (2002) interpreted the affordance effect as a manifestation of *attentional bias* toward a salient (sometimes graspable) component within an object (an attention hypothesis), not from motor codes automatically afforded by a graspable component (an affordance hypothesis). That said, the affordance hypothesis and attention hypothesis are not mutually exclusive because the time courses of the effects expected by two hypotheses can overlap. The affordance effect automatically triggered by affordance information may arise before attentional bias modulates or overrides the affordance effect. Supporting this idea, there is mounting evidence that the affordance effect could arise even without attention (Almeida, Mahon, & Caramazza, 2010; Almeida, Mahon, Nakayama, & Caramazza, 2008; Fang & He, 2005; Pappas & Mack, 2008; Yamani, Ariga, & Yamada, 2016). For example, Pappas and Mack (2008) reported that even undetected objects during the attentional blink (e.g., Shapiro, Raymond, & Arnell, 1997) potentiated compatible motor responses.

In the absence of higher cognitive processes such as attention, object affordances may boost an otherwise low signal representation of an object for more accurate perception for action. To explore this possibility, we hypothesized that object affordances facilitate the initial stage of visual processing, that is, preattentive process, with accelerated early sensory processing of affording objects. To evaluate this hypothesis, the present experiment employed a temporal order judgment task with the left- and the right-handled coffee cups as stimuli appearing with various stimulus-onset asynchronies (SOAs). A total of 16 right-handed participants judged the order of the stimulus onsets in an unspeeded manner. A point of subjective simultaneity (PSS) was computed as a measure of the difference of latencies for the perceptual entry of right-handled (more affordance) and left-handled (less affordance) stimuli.

More specifically, if object affordances speed early registry of visual information, then we predict that the onset of the right-handled cup will be perceived earlier than the onset of the left-handled cup, leading to the negative PSS for the upright cups but the PSS of approximately 0 for the inverted cups. Default Bayesian tests were used (Rouder & Morey, 2012), and Bayes factors were used as the measure of evidence for an effect of interest. Briefly, a Bayes factor indicates a ratio of likelihood that obtained data favor a statistical model including effects of interest to likelihood excluding the effects. One advantage of using Bayesian statistical approach over null-hypothesis significance tests is that the Bayesian analysis can provide evidence in favor of the null hypothesis. We report *B_10_* for Bayes factors and use terminologies for denoting the magnitude of effects as introduced in Jeffreys (1961).

## Results

**Figure 1.**
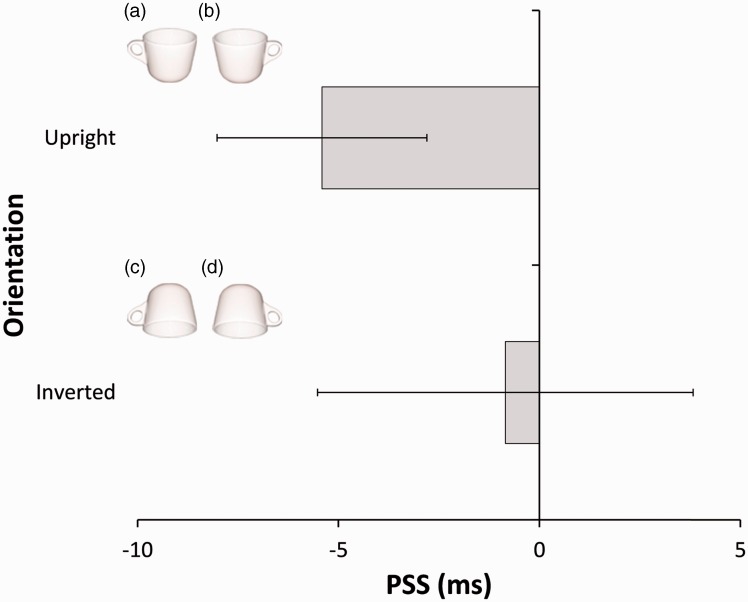
The visual stimuli and the results of the experiment. Negative PSS values indicate that the onset of the right-handled image (b, d) was perceived earlier than the onset of the left-handled image (a, c) even though the two images were presented simultaneously. Error bars denote between-subject 95% confidence intervals.

Using the psignifit program (Fründ, Haenel, & Wichmann, 2011), we calculated PSS by fitting a logistic function to the observational data and the data were analyzed using JASP (jasp-stats.org). The data consisted of the proportion of the trials wherein each participant judged the right-handled image to be presented first in each of the SOA conditions. The mean PSSs across participants for each upright and inverted condition are shown in Figure 1. Negative PSS values indicate that the right-handled image had to be presented later than the left-handled image in order for the two images to be perceived as appearing simultaneously; in other words, when the two images were presented simultaneously, the onset of the right-handled image was perceived earlier than the onset of the left-handled image. The data indicate very strong evidence that the PSS value in the upright condition was smaller than 0, one-sample *t*(15) = 4.42, *B_10_* = 70.08; the onset of the right-handled cup was perceived earlier than the onset of the left-handled cup. On the other hand, the data provide moderate evidence that the PSS in the inverted condition favored the null 0, one-sample *t*(15) = .39, *B*_10_ = 1/3.70; perceived onsets of the two cups were not different.

Consistent with the pattern of Figure 1, 93.75% of participants showed the negative PSS values in the upright condition, whereas in the inverted condition, 56.25% of participants showed the negative PSS values.

## Discussion

As expected, the onset of the right-handled stimuli was perceived earlier than that of the left-handled stimuli by 5.41 ms on average. Interestingly, this effect was observed only when the image was presented upright and the effect was eliminated with the inverted images that convey less affordance information, consistent with our prediction. Apparently, these results cannot be explained only by the attention hypothesis (Anderson et al., 2002), rather they are in favor of the affordance hypothesis, offering novel evidence concerning the affordance effect at the initial visual processing stage, or accelerated sensory processing (Shore, Spence, & Klein, 2001; Spence & Parise, 2010) of affordance objects.

One may argue that the present results are due to familiarity: Because right-handed participants presumably use right-handled cups more frequently than left-handled cups, visual experience with such visual projections might facilitate the perceptual entry of familiar objects. However, previous visual search study (Yamani et al., 2016) with the stimuli identical to those used in the present study (a right-handled target among left-handled distractors or vice versa) demonstrated the absence of a search asymmetry, for which the visual familiarity difference between a target and distractors is responsible (Rauschenberger & Yantis, 2006). Therefore, it is unlikely that visual familiarity of the right- and left-handled cup stimuli used in this study differs and is directly responsible for the effects reported here.

A number of studies have demonstrated the robustness of the affordance effect in that merely viewing depicted objects potentiates actions associated with them even though they are smaller than the actual objects (Cho & Proctor, 2010; McNair & Harris, 2014) or even though they lack in-depth information (Pappas & Mack, 2008; Pellicano, Iani, Borghi, Rubichi, & Nicoletti, 2010). Previous research further demonstrated that the effect occurs even for imaginary objects (Derbyshire, Ellis, & Tucker, 2006). That said, given the facts that an image is far from the actual object in many properties (e.g., size, color, texture, and background context) and that realistic representations of objects play an important role in producing the affordance effect (Pappas, 2014), our current results might underestimate the affordance effect; this might a bit lower the Bayes factor in this study.

According to Adamo and Ferber (2009), an object stimulus (e.g., a nail) can remain detectable during the attentional blink (e.g., Shapiro et al., 1997) when an action-related tool (e.g., a hammer) precedes the onset of the stimulus. They propose that object representations of the tool automatically trigger object affordances within the dorsal stream of visual processing (action pathway; Goodale & Milner, 1992) and that they facilitate *other* action-related objects to reach awareness. Interestingly, our findings showed that preattentively detected object affordances can recurrently facilitate their *own* object representations to gain faster access to awareness. Further investigations are warranted with regard to this issue.

In sum, object affordances are preattentively detected, in turn facilitating to form object representations by accelerating the perceptual entry processes, which may be the very foundation of the affordance effect.

## Method

A total of 16 right-handed participants (nine male; mean age = 24.00 years; mean laterality index = .87.22, SD = 15.94, Oldfield, 1971) were recruited from the community of Kyushu University. All reported normal or corrected-to-normal vision. They received 500 Japanese Yen for their participation.

The stimulus pair consisted of either of upright (Figure 1(a) and (b)) or inverted images (Figure 1(c) and (d)), which was manipulated between blocks. They were presented on a 22-in. CRT display (RDF221S; Mitsubishi, Japan). The resolution of the display was 1024 × 768 pixels and refresh rate was 100 Hz. Presenting stimuli and collecting data were computer-controlled (Mac Pro; Apple, USA). We used MATLAB with Psychtoolbox extension (Brainard, 1997; Pelli, 1997) to generate stimuli. The visual distance was 57 cm.

Participants began each trial by pressing a space bar. After the delay of a random duration between 500 and 1500 ms, a pair of stimuli, that is, left- and right-handled coffee cups (8.6° by 10.9° each), was presented on a white background with one of the variable SOAs (−70, −30, −10, 0, +10, +30, +70 ms). The stimuli were aligned vertically and separated by 11.7° above and below the central fixation. The stimulus position where each cup was presented (top or bottom) was randomized. The stimuli remained on screen until a response was given. Each stimulus pair was oriented in an upright or inverted manner (Figure 1), which was manipulated between blocks. The participants were asked to perform a two-alternative forced-choice task in which they decided which stimulus appeared first by pressing either the up or down key with their right hand. Quick responses were not encouraged. No feedback on the response accuracy was provided to the participants. The experiment consisted of two blocks, the order of which (upright first or inverted first) was counterbalanced across participants. Each experimental block consisted of 280 trials (2 stimulus positions ×7 SOAs × 20 repetitions) in a randomized order between participants. The participants performed a total of 560 trials, and the experiment took approximately 20 min.
